# Structural and Functional Changes in Prokaryotic Communities in Artificial Pit Mud during Chinese Baijiu Production

**DOI:** 10.1128/mSystems.00829-19

**Published:** 2020-03-24

**Authors:** Mao-Ke Liu, Yu-Ming Tang, Xiao-Jiao Guo, Ke Zhao, Petri Penttinen, Xin-Hui Tian, Xin-Yu Zhang, Dao-Qun Ren, Xiao-Ping Zhang

**Affiliations:** a Institute of Rice and Sorghum Sciences, Sichuan Academy of Agricultural Sciences, Deyang, People’s Republic of China; b College of Resources, Sichuan Agricultural University, Wenjiang, People’s Republic of China; University of Naples Federico II

**Keywords:** strong-ﬂavor baijiu, prokaryotic community, artificial pit mud, metaproteomics, metabolomics

## Abstract

The Chinese alcoholic beverage strong-flavor baijiu (SFB) gets its characteristic flavor during fermentation in cellars lined with pit mud. Microbes in the pit mud produce key precursors of flavor esters. The maturation time of natural pit mud of over 20 years has promoted attempts to produce artificial pit mud (APM) with a shorter maturation time. However, knowledge about the molecular basis of APM microbial dynamics and associated functional variation during SFB brewing is limited, and the role of this variability in high-quality SFB production remains poorly understood. We studied APM maturation in new cellars until the fourth brewing batch using 16S rRNA gene amplicon sequencing, quantitative PCR, metaproteomics, and metabolomics techniques. A total of 36 prokaryotic classes and 195 genera were detected. *Bacilli* and *Clostridia* dominated consistently, and the relative abundance of *Bacilli* decreased along with the APM maturation. Even though both amplicon sequencing and quantitative PCR showed increased abundance of *Clostridia*, the levels of most of the *Clostridium* proteins were similar in both the first- and fourth-batch APM samples. Six genera correlated with eight or more major flavor compounds in SFB samples. Functional prediction suggested that the prokaryotic communities in the fourth-batch APM samples were actively engaged in organic acid metabolism, and the detected higher concentrations of proteins and metabolites in the corresponding metabolic pathways supported the prediction. This multi-omics approach captured changes in the abundances of specific microbial species, proteins, and metabolites during APM maturation, which are of great significance for the optimization of APM culture technique.

**IMPORTANCE** Strong-flavor baijiu (SFB) accounts for more than 70% of all Chinese liquor production. In the Chinese baijiu brewing industry, artificial pit mud (APM) has been widely used since the 1960s to construct fermentation cellars for production of high-quality SFB. To gain insights at the systems level into the mechanisms driving APM prokaryotic taxonomic and functional dynamics and into how this variation is connected with high-quality SFB production, we performed the first combined metagenomic, metaproteomic, and metabolomic analyses of this brewing microecosystem. Together, the multi-omics approach enabled us to develop a more complete picture of the changing metabolic processes occurring in APM microbial communities during high-quality SFB production, which will be helpful for further optimization of APM culture technique and improvement of SFB quality.

## Abstract (precis)

Strong-flavor baijiu (SFB) accounts for more than 70% of all Chinese liquor production. In the Chinese baijiu brewing industry, artificial pit mud (APM) has been widely used since the 1960s to construct fermentation cellars for production of high-quality SFB. To gain insights at the systems level into the mechanisms driving APM prokaryotic taxonomic and functional dynamics and into how this variation is connected with high-quality SFB production, we performed the first combined metagenomic, metaproteomic, and metabolomic analyses of this brewing microecosystem. Together, the multi-omics approach enabled us to develop a more complete picture of the changing metabolic processes occurring in APM microbial communities during high-quality SFB production, which will be helpful for further optimization of APM culture technique and improvement of SFB quality.

## INTRODUCTION

Strong-flavor baijiu (SFB), a Chinese liquor produced by solid fermentation, is one of the oldest distillates in the world, with an annual production of 9.1 million tons (1). SFB contains over 1,300 different kinds of flavor compounds, including esters of ethyl caproate, ethyl butyrate, ethyl lactate, and ethyl acetate (2, 3). Based on the ethyl caproate content, SFB quality can be assigned into different grades, with >1.20 g liter^−1^ (4) corresponding to high quality. An increase in the ratio of ethyl caproate to ethyl lactate can enhance SFB quality (5). In the cyclical SFB production process, the anaerobic fermentation in cellars lined with pit mud lasts for 60 to 90 days (1) (see Fig. S1 in the supplemental material). The major cereal for SFB fermentation is sorghum. The fermentation involves numerous microbes originating from pit mud and a saccharifying and fermenting agent called daqu (6, 7). Microbes in the pit mud produce various small-molecule organic acids (e.g., caproic, butyric, acetic, and lactic acids) that are the key precursors of flavor esters, which largely determine the quality of SFB (1, 8, 9). The starting material for making the pit mud is fresh soil. The maturation of the pit mud over time includes the acclimation and domestication of liquor-brewing microbes during the fermentation process (10). In practice, only mature pit mud can be used to brew high-quality SFB due to a special microbial community in the mature mud (10, 11). However, the required natural maturation period of over 20 years critically restricts the production of high-quality SFB (10); the scarcity of natural mature pit mud hinders the growth and development of the baijiu industry.

10.1128/mSystems.00829-19.1 FIG S1 Workflow (a) and fermentation cellar (b) for strong-flavor baijiu (SFB) production. The major cereal for SFB fermentation is sorghum. Daqu starter is the saccharifying and fermenting agent for SFB production. Zaopei is the fermented grains. The insides of the cellar walls are lined with pit mud, providing a suitable habitat for brewing microbes that contribute to the production of flavor compounds in SFB. For the fermentation procedure, the zaopei in the lower layer is mixed with raw cereals and steamed rice hull and distilled for the SFB; then after cooling, the distilled solid residue is mixed with daqu starter and put into a cellar (normally with a volume of 6 to 8 m^3^), the top is covered with a layer of yellow mud, and the material in the cellar is allowed to ferment for 60 to 90 days. After fermentation, the zaopei in the upper layer is distilled to obtain SFB. The zaopei in the lower layer is mixed with cereals and applied in the next batch for SFB production. Download FIG S1, TIF file, 1.0 MB. Copyright © 2020 Liu et al. 2020 Liu et al. This content is distributed under the terms of the Creative Commons Attribution 4.0 International license.

Based on the knowledge on the natural pit mud microbiology, efforts to produce artificial pit mud (APM) to increase high-quality SFB production have been made since the 1960s (12). Generally, APM is prepared by inoculating a starter culture into a mixture of fresh soil, natural mature pit mud, wheat, and soybean meal, followed by incubation under anaerobic conditions for 30 to 60 days (13). This APM can be used to line the fermentation cellars, and high-quality SFB can be obtained from the first three production batches in only 1 year (14, 15), showing that a beneficial microbial community structure for SFB production can be successfully created in a short brewing period. Microbial communities in APM have been studied using a range of cultivation and molecular methods (12, 16, 17). However, our knowledge of the molecular mechanisms of APM microbial dynamics and associated functional variation and of the role of this variation in high-quality SFB production remains limited. Identifying the key microbes that drive these changes and sustain APM functionality is essential to further optimize the APM culture technique.

Recently, metagenomic analyses based on DNA sequencing have provided unprecedented profiling of microbial communities in various environments. However, this approach provides little information about the functions of the communities (18). Metaproteomics, measuring the complete protein composition expressed by a microbial community, can link proteins to specific microbes and thus provide insight into the functions and diversity of microbial communities (18). Similarly, metabolomics, characterizing the downstream products of gene expression, depicts the active metabolic pathways and provides information on their importance in biological processes (19). Together, the multi-omics approaches can provide a global perspective on microbial dynamics and their roles in driving ecosystem functions.

Our aim was to gain insights into the mechanisms driving APM prokaryotic compositional and functional dynamics during APM maturation and their connection with high-quality SFB production. The prokaryotic communities in the newly made APM and after the first four SFB brewing batches were analyzed using 16S rRNA gene amplicon sequencing, quantitative PCR (qPCR), and function prediction based on the sequencing results. To further understand the functional variation of the APM prokaryotic communities, metaproteomes and metabolomes from the first and fourth batches were analyzed using mass spectrometry (MS) techniques. Together, the multi-omics approaches enabled us to develop a more complete picture of the changing metabolic processes during APM maturation.

## RESULTS

### APM physicochemical properties.

The humus and available P contents in APM samples increased by 41.84% and 200.00% (*P* < 0.05), respectively, during APM maturation (Table 1). The moisture, organic matter, pH, total N, total P, total K, NH_4_^+^, and available K contents remained similar over time.

**TABLE 1:** The physicochemical and prokaryotic community properties in APM samples from SFB brewing process^*a*^

Parameter	Value for the parameter by brewing stage				
	Initial stage	First batch	Second batch	Third batch	Fourth batch
Moisture (%)	36.32 ± 0.20	35.96 ± 0.82	35.08 ± 1.19	34.97 ± 0.71	35.89 ± 1.50
Organic matter (%)	7.95 ± 0.14	7.74 ± 0.72	8.26 ± 1.07	9.52 ± 1.60	9.11 ± 1.19
Humus (%)	4.78 ± 0.28 B	4.93 ± 0.88 B	5.29 ± 0.93 B	5.14 ± 0.74 B	6.78 ± 0.80 A
pH	5.21 ± 0.11	5.02 ± 0.42	4.88 ± 0.49	5.72 ± 0.80	5.58 ± 0.56
Total N (g/kg)	6.61 ± 0.17	6.42 ± 0.85	6.37 ± 1.07	8.22 ± 1.51	8.31 ± 1.29
Total P (g/kg)	1.62 ± 0.10	1.63 ± 0.45	1.73 ± 0.27	2.21 ± 0.42	1.94 ± 0.46
Total K (g/kg)	16.93 ± 0.40	17.33 ± 2.08	16.67 ± 2.63	16.45 ± 2.39	15.21 ± 1.96
NH_4_^+^ (g/kg)	0.74 ± 0.07	0.87 ± 0.16	0.96 ± 0.24	0.93 ± 0.16	0.88 ± 0.25
Available P (g/kg)	0.01 ± 0 C	0.02 ± 0.01 BC	0.04 ± 0.01 A	0.02 ± 0.01 BC	0.03 ± 0.01 AB
Available K (g/kg)	2.03 ± 0.18	2.23 ± 0.35	2.14 ± 0.40	2.62 ± 0.48	2.47 ± 0.53
No. of OTUs	546.17 ± 9.57 A	420.20 ± 53.39 B	438.73 ± 26.82 B	438.63 ± 6.64 B	508.27 ± 10.84 A
Chao1	577.43 ± 16.33 A	470.61 ± 57.47 B	478.85 ± 25.55 B	464.84 ± 6.69 B	539.08 ± 28.54 A
Shannon	6.63 ± 0.50 A	3.85 ± 2.33 B	6.22 ± 0.06 A	6.83 ± 0.12 A	7.02 ± 0.11 A
Coverage (%)	99.87 ± 0.06	99.87 ± 0.06	99.87 ± 0.06	99.90 ± 0	99.90 ± 0

a Data are presented as means ± standard deviations (*n* = 3). Values with different letters within a row are significantly different statistically (*P *< 0.05). SFB, strong-flavor baijiu; APM, artificial pit mud.

### SFB flavor compounds.

**FIG 1:**
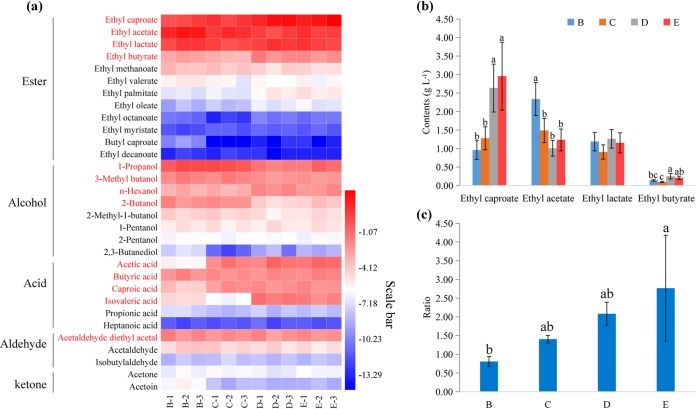
Dynamics of flavor compounds during the strong-flavor baijiu (SFB) brewing process. (a) Heat map of concentrations of flavor compounds (log_2_ scale) in SFB samples. Major compounds (average content of >0.1 g liter^−1^) presented are in red. (b) Concentrations of ethyl caproate, ethyl acetate, ethyl lactate, and ethyl butyrate in SFB samples. (c) Ethyl caproate/ethyl lactate ratio. B, first batch; C, second batch; D, third batch; E, fourth batch. In panels b and c, data are presented as means ± standard deviations (*n* = 3). Different superscript letters above bars indicate statistically significant differences (*P* < 0.05).

A total of 31 flavor compounds were detected in the SFB samples: 12 esters, 8 alcohols, 6 acids, 3 aldehydes, and 2 ketones. The major flavor compounds (average content of >0.1 g liter^−1^) included ethyl caproate, ethyl acetate, ethyl lactate, ethyl butyrate, 1-propanol, 3-methyl butanol, *n*-hexanol, 2-butanol, acetic acid, butyric acid, caproic acid, isovaleric acid, and acetaldehyde diethyl acetal. These 13 flavor compounds accounted for 95.73% of the flavor compounds present in the samples (Fig. 1a; see Table S1 in the supplemental material).

10.1128/mSystems.00829-19.2 TABLE S1 Concentrations (g liter^−1^) of flavor compounds in strong-flavor baijiu samples. Download Table S1, XLSX file, 0.01 MB. Copyright © 2020 Liu et al. 2020 Liu et al. This content is distributed under the terms of the Creative Commons Attribution 4.0 International license.

Among esters, the contents of ethyl caproate and ethyl butyrate increased during the brewing process (*P < *0.05), reaching maximum levels in the fourth and third batches of SFB samples, respectively. The ethyl acetate content decreased significantly from the first to the later batches (*P <* 0.05) (Fig. 1b). The ratio of ethyl caproate/ethyl lactate increased steadily from 0.81 to 2.77 (*P <* 0.05), indicating an improvement in the quality of SFB (Fig. 1c).

### Amplicon sequencing and qPCR of 16S rRNA genes.

**FIG 2:**
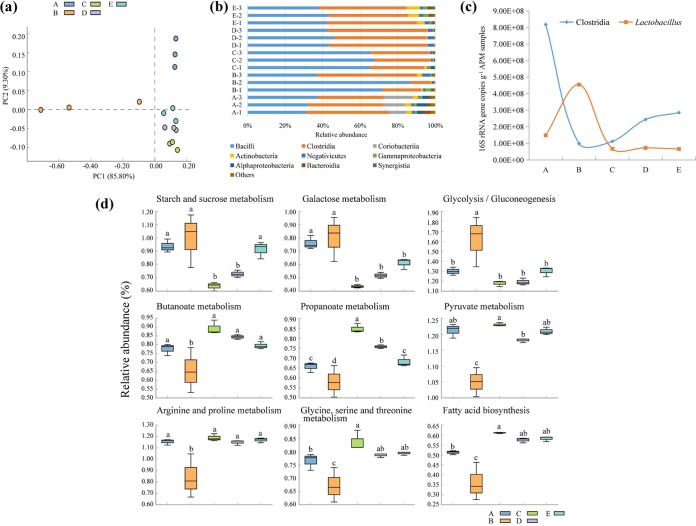
16S rRNA gene analysis of prokaryotic communities in artificial pit mud (APM) samples. (a) Principal-component analysis (PCA) at the operational taxonomic unit (OTU) level. (b) Proportions of the most abundant prokaryotic classes. (c) Average number (*n* = 3) of 16S RNA gene copies of *Clostridia* and *Lactobacillus*. (d) Predicted abundances of selected KEGG orthologs (KOs) related to saccharides and organic acid metabolisms. A, initial stage; B, first batch; C, second batch; D, third batch; E, fourth batch. Different superscript letters indicate statistically significant differences (*P < *0.05).

The 1,241,812 sequences that passed quality filtering were binned into 420.20 to 546.17 operational taxonomic units (OTUs) per APM maturation stage (Table 1). The coverage was ≥99.8%, indicating that the samples represented well the sampled prokaryotic communities. The Chao1 index was lower in samples from the first to third batch than in those of the initial and fourth batches (*P <* 0.05). The Shannon index was lower in samples from the first batch than in the other samples (*P <* 0.05) (Table 1). In the principal-component analysis (PCA), the prokaryotic communities showed batch-dependent clustering, with the exception of the first-batch APM samples, which were scattered along the PCA axis 1 (Fig. 2a).

The OTUs were classified into 36 prokaryotic classes. The nine most prevalent classes, with average abundances of >1% in at least one APM maturation stage, were *Bacilli*, *Clostridia*, *Coriobacteriia*, *Actinobacteria*, *Negativicutes*, *Gammaproteobacteria*, *Alphaproteobacteria*, *Bacteroidia*, and *Synergistia* (Fig. 2b). *Bacilli* and *Clostridia* were the only classes that consistently dominated throughout the whole brewing process, representing 31.37 to 86.60% and 11.16 to 53.33%, respectively, of the total abundances. The relative abundance of *Clostridia* was higher in the third batch than in the first and second batches, whereas the relative abundances of *Bacilli* were higher in the first and second batches than in the other stages (*P <* 0.05). The relative abundances of *Coriobacteriia* and *Alphaproteobacteria* were highest in the initial stage, and those of *Actinobacteria* and *Synergistia* were highest in the fourth batch (*P <* 0.05) (Table 2).

**TABLE 2:** Prokaryotic taxa with relative abundances of >1% in at least one stage in APM samples from the SFB brewing process^*a*^

Class or genus	Relative abundance by brewing step				
	Initial stage	First batch	Second batch	Third batch	Fourth batch
*Clostridia*	39.77 ± 4.50 AB	28.41 ± 22.11 B	29.83 ± 1.31 B	51.65 ± 1.98 A	45.61 ± 2.94 AB
* Clostridium*	12.81 ± 2.20 CD	8.53 ± 6.07 D	14.5 ± 0.27 BC	26.41 ± 0.84 A	19.82 ± 1.72 B
* Caproiciproducens*	9.58 ± 2.77 A	0.47 ± 0.48 C	0.53 ± 0.22 C	0.94 ± 0.24 C	6.58 ± 2.29 B
* Hydrogenispora*	2.31 ± 0.46	4.81 ± 5.03	1.03 ± 0.12	2.37 ± 0.38	2.04 ± 0.14
* Sedimentibacter*	1.28 ± 0.36	1.52 ± 1.32	0.63 ± 0.12	1.10 ± 0.15	0.70 ± 0.16
* Tepidimicrobium*	0.90 ± 0.41	1.09 ± 0.91	1.09 ± 0.26	0.33 ± 0.01	0.24 ± 0.06
* Garciella*	1.34 ± 0.40 A	0.25 ± 0.22 B	1.70 ± 0.23 A	0.19 ± 0.04 B	0.10 ± 0.02 B
* Anaerosporobacter*	0.47 ± 0.28 B	0.37 ± 0.32 B	0.73 ± 0.09 AB	1.06 ± 0.18 A	0.69 ± 0.10 AB
* Caldicoprobacter*	0.93 ± 0.38	1.27 ± 1.12	0.26 ± 0.09	0.27 ± 0.05	0.23 ± 0.05
* Anaerosalibacter*	0.47 ± 0.16 B	0.62 ± 0.51 B	1.48 ± 0.13 A	0.22 ± 0.04 B	0.18 ± 0.03 B
* Desulfosporosinus*	0.18 ± 0.03 C	0.35 ± 0.27 C	0.24 ± 0.06 C	1.14 ± 0.11 A	0.83 ± 0.08 B
* Desulfitobacterium*	0.10 ± 0.02 C	0.21 ± 0.10 C	0.25 ± 0.10 C	1.28 ± 0.04 A	1.02 ± 0.15 B
* Fonticella*	0.13 ±0.05 B	0.14 ± 0.05 B	0.13 ± 0.05 B	1.42 ± 0.53 A	1.02 ± 0.30 A
* Haloimpatiens*	0.04 ± 0.01 B	0.03 ± 0.02 B	0.06 ± 0.03 B	1.17 ± 0.06 A	0.98 ± 0.33 A
*Bacilli*	33.52 ± 3.21 B	65.07 ± 25.51 A	66.15 ± 1.22 A	43.93 ± 1.77 B	41.22 ± 2.78 B
* Lactobacillus*	19.87± 5.09 B	57.67 ± 31.45 A	3.13 ± 1.74 B	2.80 ± 3.74 B	4.23 ± 4.09 B
* Bacillus*	4.84 ± 1.72 B	3.12 ± 2.54 B	10.84 ± 1.19 A	8.52 ± 0.34 A	7.99 ± 1.12 A
* Hazenella*	0.26 ± 0.11 C	0.25 ± 0.14 C	9.94 ± 1.16 A	9.50 ± 0.70 A	5.96 ± 1.61 B
* Weissella*	0.68 ± 0.35 B	0.01 ± 0.01 B	0.03 ± 0.03 B	0 ± 0 B	3.71 ± 1.30 A
* Paenibacillus*	0.83 ± 0.58 D	0.27 ± 0.23 D	5.90 ± 0.16 A	3.19 ± 0.48 B	2.45 ± 0.27 C
* Kroppenstedtia*	0.45 ± 0.10 D	0.63 ± 0.42 D	6.61 ± 0.46 A	1.92 ± 0.30 B	1.24 ± 0.20 C
* Lactococcus*	0.67 ± 0.20 B	0 ± 0 C	0 ± 0 C	0 ± 0 C	1.27 ± 0.44 A
* Baia*	0.11 ± 0.04 C	0.17 ± 0.10 C	4.15 ± 1.03 A	3.50 ± 0.23 A	2.17 ± 0.59 B
* Rummeliibacillus*	0.41 ± 0.16 C	0.25 ± 0.20 C	1.15 ± 0.17 B	1.80 ± 0.22 A	1.43 ± 0.28 AB
* Brevibacillus*	0.23 ± 0.08 C	0.16 ± 0.10 C	2.06 ± 0.63 A	1.22 ± 0.20 B	1.10 ± 0.24 B
* Lysinibacillus*	0.24 ± 0.02	0.11 ± 0.06	1.15 ± 1.31	1.11 ± 0.08	0.74 ± 0.11
* Pediococcus*	1.86 ± 0.66 A	0.11 ± 0.17 B	0 ± 0 B	0 ± 0 B	0.08 ± 0.02 B
* Novibacillus*	0.02 ± 0.01 C	0.04 ± 0.03 C	1.26 ± 0.12 A	0.22 ± 0.02 B	0.17 ± 0.02 B
*Actinobacteria*	3.26 ± 0.18 B	1.29 ± 1.26 C	0.74 ± 0.59 C	0.37 ± 0.14 C	5.60 ± 1.38 A
* Neoscardovia*	0.07 ± 0.02 B	0 ± 0 B	0 ± 0 B	0 ± 0 B	4.60 ± 1.55 A
* Atopobium*	11.57 ± 3.57 A	0.10 ±0.10 B	0.07 ± 0.07 B	0 ± 0 B	0.01 ± 0.01 B
*Synergistia*	0.05 ± 0.02 B	0.12 ± 0.18 B	0.003 ± 0.006 B	0.003 ± 0.006 B	1.05 ± 0.50 A
* Aminobacterium*	0.04 ± 0.03 B	0.11 ± 0.17 B	0.003 ± 0.006 B	0.003 ± 0.006 B	1.04 ± 0.49 A
*Bacteroidia*	2.40 ± 1.36 A	0.33 ± 0.50 B	0.24 ± 0.20 B	0.09 ± 0.11 B	1.21 ± 0.46 AB
* Bacteroides*	2.02 ± 1.18 A	0.007 ± 0.006 B	0.003 ± 0.006 B	0.03 ± 0.04 B	0.02 ± 0.02 B
*Coriobacteriia*	12.11 ± 3.28 A	0.11 ± 0.11 B	0.08 ± 0.07 B	0 ± 0 B	0.11 ± 0.05 B
*Negativicutes*	1.16 ± 0.29 B	2.34 ± 1.90 AB	0.94 ± 0.38 B	3.08 ± 0.38 A	2.14 ± 0.42 AB
*Gammaproteobacteria*	1.42 ± 0.19 AB	1.43 ± 1.37 AB	0.86 ± 0.35 AB	0.55 ± 0.16 B	2.17 ± 0.90 A
*Alphaproteobacteria*	4.03 ± 1.18 A	0.08 ± 0.10 B	0.81 ± 1.19 B	0.03 ± 0.02 B	0.16 ± 0.06 B

a Data are presented as means ± standard deviations (*n* = 3). Values with different letters within a row are significantly different statistically (*P *< 0.05). SFB, strong-flavor baijiu; APM, artificial pit mud.

A total of 195 genera were detected (Table S2), out of which 30 genera dominated the APM prokaryotic community, representing 63.00 to 93.41% of the total abundances (Table 2). In the *Clostridia*, the relative abundances of the initially rare *Desulfosporosinus*, *Fonticella*, and *Haloimpatiens* were higher in the third and fourth batches than in the other stages, and those of *Clostridium* were higher in the third and fourth batches than in the initial stage and first batch (*P <* 0.05). The relative abundance of *Lactobacillus* in the first batch was almost three times higher than that in the initial stage and over 10 times higher than that in the subsequent batches (*P <* 0.05). The relative abundances of eight other genera in the class *Bacilli* were higher in the second, third, and fourth batches than in the initial stage and first batch, and those of *Weissella* and *Lactococcus* were highest in the fourth batch, similar to abundances of *Neoscardovia* (class *Actinobacteria*) and *Aminobacterium* (class *Synergistia*) (*P <* 0.05). *Pediococcus* (*Bacilli*), *Atopobium* (*Actinobacteria*), and *Bacteroides* (class *Bacteroidia*) were abundant in the initial stage but mostly depleted in the subsequent brewing process (*P <* 0.05) (Table 2).

10.1128/mSystems.00829-19.3 TABLE S2 Relative proportion (%) of the prokaryotic communities at the genus level in artificial pit mud (APM) samples. Download Table S2, XLSX file, 0.03 MB. Copyright © 2020 Liu et al. 2020 Liu et al. This content is distributed under the terms of the Creative Commons Attribution 4.0 International license.

The most abundant taxa, *Clostridia* and *Lactobacillus*, were quantified using qPCR (Fig. 2c). The concentration of *Clostridia* decreased significantly from an initial 8.18 × 10^8^ to 9.83 × 10^7^ copies g^−1^ measured at the first batch and then increased to 2.85 × 10^8^ copies g^−1^ at the end of the fourth batch. In line with the changes in relative abundances, *Lactobacillus* abundance increased from the initial stage to the first batch, reaching a maximum value of 4.54 × 10^8^ copies g^−1^, and was approximately 7 × 10^7^ copies g^−1^ in the subsequent batches.

### Relationships of prokaryotic community with physicochemical properties and major SFB flavor compounds.

**FIG 3:**
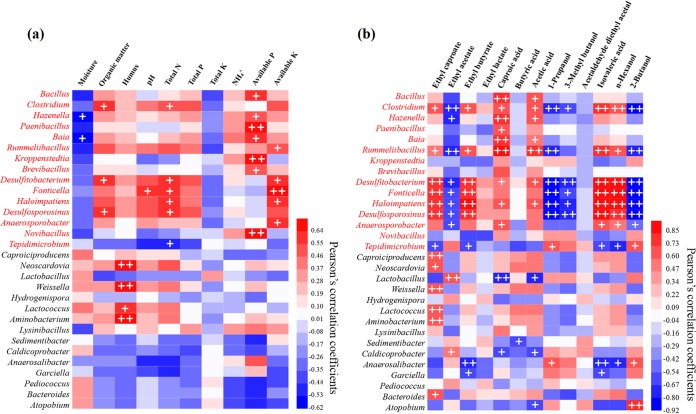
Pearson’s correlation coefficients between dominant prokaryotic genera and physicochemical properties of APM samples (a) and flavor components of SFB samples (b). +, correlation significant at a *P *value of *<*0.05; ++, correlation significant at a *P *value of *<*0.01. The genera significantly correlated with total N, available K, and available P are indicated with a red font.

The Pearson’s correlation analysis between APM physicochemical properties and most abundant prokaryotic genera showed that total N, available P, and available K correlated with 15 genera (Fig. 3a). Out of the 15 genera, 6 genera correlated with eight or more major flavor compounds in SFB samples (Fig. 3b). *Clostridium*, *Rummeliibacillus*, *Desulfitobacterium*, *Fonticella*, *Haloimpatiens*, and *Desulfosporosinus* correlated positively with ethyl caproate, ethyl butyrate, isovaleric acid, and *n*-hexanol, and *Clostridium*, *Rummeliibacillus*, *Desulfitobacterium*, and *Haloimpatiens* correlated positively with caproic acid and acetic acid (*P <* 0.05). The six genera correlated negatively with ethyl acetate, 1-propanol, and 2-butanol, and all six, except *Rummeliibacillus*, correlated negatively with 3-methyl butanol (*P <* 0.05).

### Predicted metabolic profile of the prokaryotic community.

The PICRUSt (Phylogenetic Investigation of Communities by Reconstruction of Unobserved States) software package identified 279 functional categories in the Kyoto Encyclopedia of Genes and Genome (KEGG) pathways. We focused on the predicted abundances of KEGG orthologs (KOs) assigned to saccharide and organic acid metabolism. Generally, the relative abundances of KOs were at the same levels in the initial stage and fourth batch, with the exception of galactose metabolism KOs (Fig. 2d). The relative abundances of KOs assigned to the starch and sucrose metabolism were highest in the initial stage and first and fourth batches, those of galactose metabolism KOs were highest in the initial stage and first batch, and those of glycolysis/gluconeogenesis KOs were highest in the first batch (*P <* 0.05). The relative abundances of KOs assigned to organic acid metabolism (butanoate metabolism, propanoate metabolism, pyruvate metabolism, arginine and proline metabolism, fatty acid biosynthesis, and glycine, serine, and threonine metabolism) were lowest in the first batch (*P <* 0.05) (Fig. 2d).

### Metaproteomic analysis of APM prokaryotic communities.

Based on the differences in the ratios of ethyl caproate to ethyl lactate, an SFB quality measure, and PICRUSt predictions, we next performed metaproteomics and metabolomics analyses of the first- and fourth-batch APM samples to obtain more insight into the functional changes and their relation to the SFB quality.

**FIG 4:**
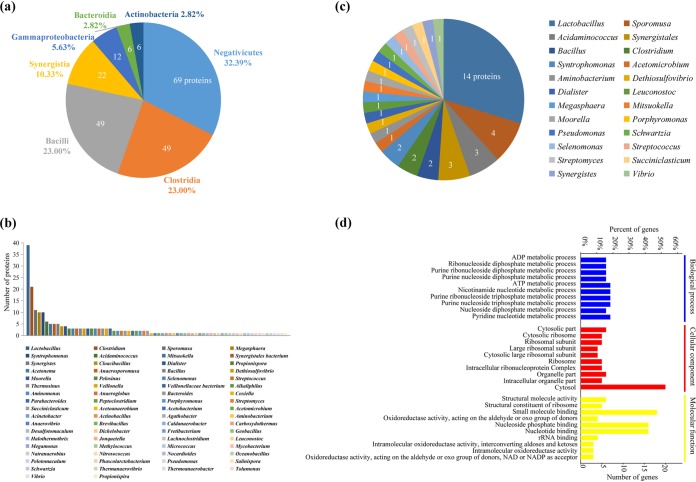
Metaproteomic analysis of prokaryotic communities in artificial pit mud (APM) samples of the first and fourth batches. Prokaryotic classification based on the identified proteins at the class (a) and the genus (b) levels. (c) Sources of differential proteins. (d) GO enrichment for differential proteins in biological process, cellular component, and molecular function categories.

The peptide spectra identified by a tandem mass tags (TMT)-MS proteomics approach were searched against a prokaryotic database created by collating all UniProt entries (a total of 610,836 entries) from organisms with an abundance of >1% in the 16S rRNA gene amplicon sequences identified in the first- and fourth-batch APM samples, including *Bacilli*, *Clostridia*, *Actinobacteria*, *Synergistia*, *Negativicutes*, *Gammaproteobacteria*, and *Bacteroidia* (Table 2). A total of 213 prokaryotic proteins were identified. The three most abundant classes collectively accounted for 78.39% of prokaryotic proteins (Fig. 4a). *Negativicutes* (32.39%) was the most abundant class, followed by *Clostridia* (23.00%) and *Bacilli* (23.00%). The proteins originated from 70 genera; 18.31% were from *Lactobacillus*, 9.86% were from *Clostridium*, and 5.16% were from *Sporomusa* (Fig. 4b). A total of 47 differential proteins with a fold change of >2.0 or <0.50 (*P* < 0.05) were found; 16 and 31 proteins were enriched and depleted, respectively, in the fourth-batch APM samples (Table S3). The differential proteins originated from 24 genera and mainly belonged to *Lactobacillus* (Fig. 4c). In line with the changes in 16S rRNA gene amplicon relative abundances and qPCR results, the 14 detected *Lactobacillus* proteins were depleted in the fourth-batch APM samples (Table S3).

10.1128/mSystems.00829-19.4 TABLE S3 Differential proteins in the artificial pit mud (APM) samples (fourth batch/first batch). Download Table S3, XLSX file, 0.02 MB. Copyright © 2020 Liu et al. 2020 Liu et al. This content is distributed under the terms of the Creative Commons Attribution 4.0 International license.

To validate our metaproteomic data, 15 differentially expressed proteins, based on the Sequest HT score (Table S3), were selected, and their protein levels were measured by a parallel reaction monitoring (PRM)-MS proteomics approach. The fold changes of the proteins between the first- and fourth-batch APM samples were consistent with those determined by TMT-MS analysis (Table 3).

**TABLE 3:** Comparison of the fourth-batch and first-batch APM samples from the strong-flavor baijiu brewing process by TMT- and PRM-based quantitative proteomics approaches^*a*^

Accession no.^*c*^	Protein name	Gene symbol	Fold change by:^*b*^		Protein source
			TMT	PRM	
A0A1M4T8Q5	Cell division protein FtsZ	*ftsZ*	0.46	0.48*	Schwartzia succinivorans
A5N5B3	Formate-tetrahydrofolate ligase	*fhs*	0.48	0.20*	Clostridium kluyveri
A7Z207	60-kDa chaperonin	*groL*	0.39	0.26*	Bacillus velezensis
A8YUI5	Glucose-6-phosphate isomerase	*pgi*	0.35	0.42*	Lactobacillus helveticus
B1MW06	50S ribosomal protein L29	*rpmC*	0.34	0.15*	Leuconostoc citreum
C9LNB3	Methylmalonyl-CoA mutase domain protein	*GCWU000321_01033*	2.10	8.06*	Dialister invisus
D5EGB9	S-layer domain protein	*Amico_1484*	3.85	82.97*	Aminobacterium colombiense
O32755	Glyceraldehyde-3-phosphate dehydrogenase	*gap*	0.34	0.29*	Lactobacillus delbrueckii subsp. *bulgaricus*
O32765	l-Lactate dehydrogenase	*ldh*	0.40	0.25*	Lactobacillus helveticus
P80583	Flagellin (fragment)	*fla*	3.71	9.53*	Clostridium tyrobutyricum
Q0AVM3	Acetyl-CoA acetyltransferase	*Swol_1934*	2.16	8.61*	Syntrophomonas wolfei subsp. *wolfei*
Q0AZ33	Electron transfer flavoprotein subunit alpha	*etfA*	2.02	10.65*	Syntrophomonas wolfei subsp. *wolfei*
Q74IT6	Chaperone protein DnaK	*dnaK*	0.37	0.21*	Lactobacillus johnsonii
Q7MVY0	Hydroxylamine reductase	*hcp*	0.43	0.12*	Porphyromonas gingivalis
R7KAP8	Acetate kinase	*ackA*	2.41	573.92*	*Acidaminococcus* sp. CAG:917

a APM, artificial pit mud; TMT, tandem mass tag; PRM, parallel reaction monitoring.

b The asterisks indicate statistically significant differences between values of the fourth batch and those of the first-batch APM samples (*P *< 0.05).

c NCBI or UniProt.

The differentially expressed proteins were submitted for Gene Ontology (GO) enrichment analysis (Fig. 4d). In biological process (BP) analysis, the greatest changes occurred in metabolic processes, including ADP metabolic process, purine ribonucleoside diphosphate metabolic process, and purine nucleoside diphosphate metabolic process. Cellular component (CC) analysis showed that most of the proteins acted in the cytosol. The molecular function (MF) analysis revealed that most proteins were involved in small-molecule binding, nucleoside phosphate binding, and nucleotide binding.

### Metabolomic analysis of APM.

**FIG 5:**
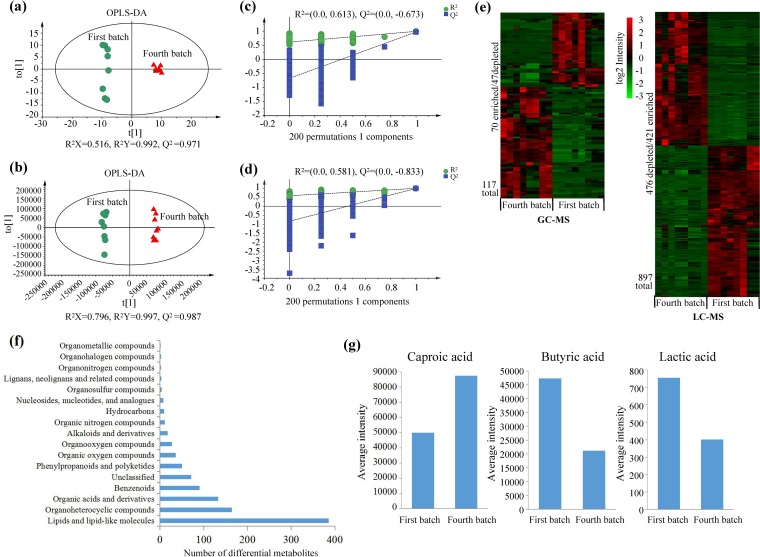
Metabolomic analysis of the artificial pit mud (APM) samples of the first and fourth batches (*n* = 8). Orthogonal partial least-squares discriminant analysis (OPLS-DA) scatter plots (a and b) and the corresponding cross-validation plots (c and d) derived from gas chromatography-mass spectrometry (GC-MS) (a and c) and liquid chromatography-mass spectrometry (LC-MS) (b and d) data sets. Intensity levels (e) and functional groups (f) of differential metabolites are shown. (g) Average levels of caproic, butyric, and lactic acids. For panels a to d, the *R*^2^*X* and *R*^2^*Y* values show the total amount of variation explained by the model in the *X* and *Y* matrix, respectively. The *Q*^2^ value represents the predictive ability of the models.

In the untargeted metabolomics analysis, we identified 7,478 metabolites, out of which 246 were identified by gas chromatography (GC)-MS, and 7,232 were identified by liquid chromatography (LC)-MS from the first- and fourth-batch APM samples. Both GC-MS and LC-MS approaches led to a clear separation of the batches in the orthogonal partial least-squares discriminant analysis (OPLS-DA) scatter plots, and based on cross-validation, the predictive ability and goodness of fit of the OPLS-DA models were credible (Fig. 5a to d). In line with PCA of the prokaryotic communities (Fig. 2a), the metabolic profiles from the first batch were more dispersed than those from the fourth batch. A total of 1,014 metabolites (117 detected by GC-MS and 897 detected by LC-MS) were identified as differential (variable importance in projection [VIP] of >1; *P <* 0.05). A total of 491 and 523 metabolites were enriched and depleted, respectively, in the fourth-batch APM samples (Fig. 5e; Table S4). The differential metabolites were clustered into 16 functional classes (Fig. 5f). Lipids and lipid-like molecules (38.07%) were the most prevalent, followed by organoheterocyclic compounds (16.17%) and organic acids and derivatives (13.11%).

10.1128/mSystems.00829-19.5 TABLE S4 List of differential metabolites measured by gas chromatography-mass spectrometry (GC-MS) and liquid chromatography-mass spectrometry (LC-MS). Download Table S4, XLSX file, 0.1 MB. Copyright © 2020 Liu et al. 2020 Liu et al. This content is distributed under the terms of the Creative Commons Attribution 4.0 International license.

### Metabolic pathways related to the synthesis of key substance-determining flavor esters in SFB.

**FIG 6:**
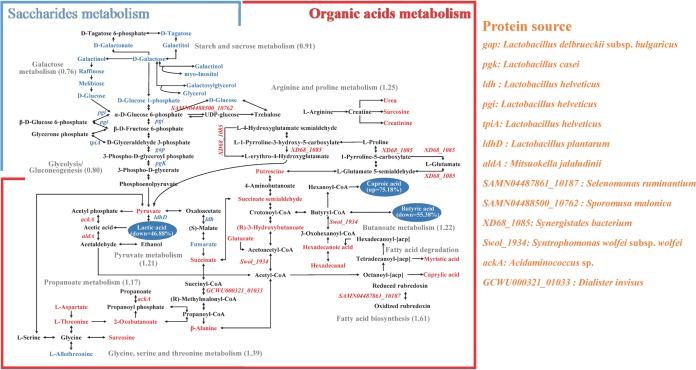
Schematic presentation of pathways, proteins, metabolites, and species related to the synthesis of caproic, butyric, and lactic acids in artificial pit mud (APM) samples. The pathways were organized based on the Escherichia coli K-12 MG 1655 genome with KEGG (Kyoto Encyclopedia of Genes and Genomes) organism identifier *eco* (http://www.kegg.jp/). Metabolites and proteins that are enriched or depleted in the fourth-batch APM samples compared to levels in the first-batch APM samples are indicated in red and blue, respectively. The average fold changes of the predicted abundances of KEGG orthologs (KOs) based on the 16S rRNA gene amplicon sequencing (Fig. 2d) are indicated in parentheses.

Out of the key substance-determining flavor esters in SFB, the levels of caproic acid were higher in the fourth-batch APM samples than in the first-batch APM samples, and those of butyric and lactic acid were lower (*P >* 0.05) (Fig. 5g). Based on KEGG enrichment analysis, the metabolic pathways involved in the production of caproic, butyric, and lactic acids were determined (Fig. 6; Table S5). The multiple pathways are in species belonging to *Lactobacillus*, *Mitsuokella*, *Selenomonas*, *Sporomusa*, *Syntrophomonas*, *Acidaminococcus*, *Dialister*, and *Synergistales*.

10.1128/mSystems.00829-19.6 TABLE S5 Pathways, differential metabolites and proteins, and species related to the synthesis of caproic, butyric, and lactic acids. Download Table S5, XLSX file, 0.02 MB. Copyright © 2020 Liu et al. 2020 Liu et al. This content is distributed under the terms of the Creative Commons Attribution 4.0 International license.

Within the saccharide metabolism pathway, except for the putative aldehyde dehydrogenase AldA (gene symbol *aldA*) and ethanolamine utilization protein EutM (*SAMN04488500_10762*), most proteins were depleted in the fourth-batch APM samples. Except for pyruvate, the remaining 12 metabolites were also depleted. In contrast, except for d-lactate dehydrogenase (*ldhD*) and l-lactate dehydrogenase (*ldh*), most proteins involved in the metabolism of organic acids were enriched. Consistently, except for fumarate and l-allothreonine, the remaining 17 metabolites were also enriched in the fourth-batch APM samples. The changes were in agreement with the results of PICRUSt predictive metagenome analysis (Fig. 2d).

Among the multiple pathways, butanoate metabolism and pyruvate metabolism pathways are directly involved in the production of caproic acid, butyric acid, and lactic acid. In pyruvate metabolism, the levels of three proteins, acetyl-coenzyme A (CoA) acetyltransferase (*Swol_1934*), acetate kinase (*ackA*), and *aldA*, and two metabolites (pyruvate and succinate) were enriched, while two proteins (*ldhD* and *ldh*) and one metabolite (fumarate) were depleted in the fourth-batch APM samples. In butanoate metabolism, except for fumarate, the remaining metabolites (pyruvate, succinate, succinate semialdehyde, and *R*-3-hydroxybutanoate) and the protein *Swol_1934* were enriched in the fourth-batch APM samples.

## DISCUSSION

Strong-flavor baijiu (SFB) is a fermented and distilled alcoholic beverage that is fermented in production cellars lined with pit mud, a fresh soil derivative with characteristic microbial communities. Pit mud affects both the quality and safety of the end product. The maturation time of natural pit mud of over 20 years and the stochastic nature of maturation have promoted attempts to produce artificial pit mud (APM) with shorter and more predictable maturation (13). We studied APM maturation and its connection with high-quality SFB production by assessing the composition and functions of the APM prokaryotic community using combined metagenomics, metaproteomics, and metabolomics.

The major flavor compounds of SFB are ethyl caproate balanced with ethyl lactate, ethyl acetate, and ethyl butyrate (3). Ethyl caproate, produced from the esterification of caproic acid and ethanol, is a key compound affecting SFB quality (4). The ethyl caproate contents of SFB obtained from a new APM-lined cellar increased from 2.24 g liter^−1^ in the first batch to 5.24 g liter^−1^ in the third batch (14). Although the ethyl caproate contents of our SFB samples were lower, the contents increased during the APM maturation, and both the ethyl caproate content and ethyl caproate/ethyl lactate ratio reached levels required for high-quality SFB (4, 5). Hence, the acquired amplicon sequencing, metaproteomics, and metabolomics data are relevant in studying the role of APM microbial dynamics in high-quality SFB production.

Amplicon sequencing targeting the 16S rRNA gene revealed that after the first batch of fermentation, the prokaryotic community was less diverse and more dispersed than that after the subsequent batches, possibly due to niche selection (10). In the early stage of brewing, the species that could not adapt to the brewing environment were eliminated, resulting in the differentiation of microbial community structure. The subsequent adaptation of brewing microbes to the environment may partially explain the increased species diversity and stability in the community. Interestingly, the changes were similar to those observed in 1-, 10-, 25-, and 50-year-old natural pit muds (10), suggesting that the maturation processes in both natural and artificial pit muds proceed similarly yet at a different pace. APM is degraded relatively frequently, leading to poor SFB quality (17), and this may be an unavoidable consequence of the quicker maturation process.

In earlier studies, *Clostridium* and *Hydrogenispora* were predominant microbes in natural mature pit mud (20, 21), *Bacillus* was predominant in immature pit mud (1), and the *Clostridia*/*Bacilli* ratio correlated positively with pit mud quality (22). Similarly, in our study, the relative abundances of the classes *Bacilli* and *Clostridia* and the genera *Clostridium*, *Hydrogenispora*, *Lactobacillus*, and *Bacillus* changed with APM age. Again, similar to characteristics of the natural pit mud (10), the relative abundances of *Clostridia* increased, and those of *Bacilli* decreased along with the APM maturation. Lactic acid bacteria have been reported to be predominant in both new and degraded pit mud (17, 22), and the quantification of *Lactobacillus* 16S rRNA genes has been applied to evaluate the state of pit mud (23). In agreement, both the relative abundance and gene copy numbers of *Lactobacillus* were highest in the first-batch APM samples. The dominance of *Lactobacillus* was the likely reason for the low diversity at that stage; possibly the early-stage brewing environment was favorable for *Lactobacillus* (10). The subsequent changes in the environment may at least partially explain the observed increase in species diversity of APM. Among the genera that increased during the APM maturation, the increase of *Fonticella*, *Haloimpatiens*, and *Desulfosporosinus* organisms might be related to their slow growth as strictly anaerobic bacteria (24–26). However, the genera that increased included also facultatively anaerobic bacteria, for example *Weissella* (27). To our knowledge, *Fonticella* and *Hazenella* were detected for the first time in SFB production, suggesting that the APM microbial communities and their succession are more complex than previously known (12, 16, 17).

Interestingly, compared with communities identified in natural mature pit mud (10, 28), the abundances of methanogens were very low in our study. For example, the average abundance of *Methanobrevibacter* was 1.94 to 14.34% in natural mature pit mud samples (10, 28) but less than 0.05% in our APM samples. *Methanobrevibacter* is a hydrogenotrophic methanogen that produces CH_4_ from H_2_ and CO_2_ (29). Together with fermentative bacteria, methanogens can enhance organic acid production through syntrophic interactions (30). Thus, the high abundances of *Methanobrevibacter* in natural mature pit mud may indicate efficient organic acid production, which can increase flavor ester formation during SFB production. Further studies are required to determine the contribution of the differences in microbial community structure between artificial and natural pit mud to SFB production.

Physicochemical properties play an important role in shaping microbial community structure and richness in the environment, including brewing ecosystems (10, 31). During long-time, natural maturation processes, the changes in pit mud microbial community composition were related to changes in pH and NH_4_^+^ content (10). In our study, the genera *Clostridium*, *Rummeliibacillus*, *Desulfitobacterium*, *Fonticella*, *Haloimpatiens*, and *Desulfosporosinus* correlated positively with total N, available P, and available K contents. These genera correlated positively with the flavor compounds ethyl caproate, ethyl butyrate, and caproic acid and negatively with 3-methyl butanol and 2-butanol. The accumulation of fusel alcohols such as 3-methyl butanol and 2-butanol in SFB may have toxic effects, such as nervous hyperemia, dizziness, and headaches (32). This may suggest that an appropriate increase in the contents of total N, available K, and available P in APM can improve overall SFB flavor and the degree of comfort and satisfaction after drinking. Interestingly, except for *Clostridium*, which can produce caproic acid, the major metabolites of the other dominant genera are not directly related to the flavor compounds. For example, *Rummeliibacillus* produces indole (33), *Fonticella* and *Haloimpatiens* ferment saccharides to formate, acetate, lactate, ethanol, and CO_2_ (24, 25), *Desulfitobacterium* ferments pyruvate to lactate and acetate (34), and *Desulfosporosinus* includes species able to produce acetate (26). However, the significant correlation between these genera and the SFB flavor compounds may indicate that the APM microbes have indirect roles and synergistic effects in the flavor compound formation.

Compared with the proteins previously detected from soil (35), the number of prokaryotic proteins (a total of 213 proteins) detected from the APM in this study was low. In addition to the different nature of the samples, the selection of the database for protein identification may affect the number of identified proteins (35, 36). For metaproteomic analysis, one particular challenge is the choice of an appropriate database for peptide matching (37). For example, we initially identified the proteins through the UniProt database covering bacteria, archaea, and fungi and obtained a total of 324 microbial proteins (data not shown). However, to identify proteins more accurately, based on the sequencing data, a database was generated by collating entries in the UniProt database covering the dominant bacterial classes (>1%) in the first- and fourth-batch APM samples. This enabled us to limit the size of the database, which can reduce the search space and decrease the potential for false positives, and to allow the peptides to be matched against genomic data that are specific to the same taxa (37–39). In general, the results show good consistency between the amplicon sequencing and the metaproteomic data with respect to taxonomic structure. For example, *Clostridium* and *Lactobacillus* were abundant in both analyses. Consistent with the observed decrease in the relative abundance and gene copy number of *Lactobacillus*, the levels of *Lactobacillus* proteins were lower in the fourth-batch APM than in the first-batch APM. However, there were outliers. For example, *Syntrophomonas* contributed less than 0.01% to the 16S rRNA gene community, and *Hazenella* and *Weissella* contributed almost 6% and 4%, respectively; yet in the metaproteome more than 4% of all proteins were assigned to *Syntrophomonas*, and none were assigned to *Hazenella* and *Weissella*. Furthermore, even though both amplicon sequencing and qPCR showed increased abundance of *Clostridia*, the levels of most of the *Clostridium* proteins were similar in both the first- and fourth-batch APM samples. The reasons for these conflicting results may include some of the following: (i) the presence of DNA from dead organisms (40); (ii) bias in the DNA extraction, PCR, sequencing, and bioinformatics steps in amplicon sequencing (41); (iii) expression of the proteins below the threshold of detection at the time of sampling (39); or (iv) underrepresentation of the proteins in the protein sequence database (35). The last two points may explain the small number of proteins identified in this study. Together, these conflicting results add further evidence to previous findings that phylogenetic composition alone cannot reflect changes in the functions expressed by bacterial communities (42), highlighting the importance of multi-omics study designs to reveal the effects of brewing microbial communities and their contribution to SFB production.

To illustrate the main effects of the microbial dynamics in APM on SFB production, the multi-omics data were summarized in an overview model. Functional prediction suggested that the prokaryotic communities in the fourth-batch APM samples were actively engaged in organic acid metabolism, and the detected higher concentrations of proteins and metabolites in the corresponding metabolic pathways supported the prediction. Theoretically, the predicted enrichment of butanoate metabolism may be favorable for butyric acid production, providing more precursors for caproic acid synthesis (21, 43). We speculate that the decreased levels of butyric acid might result from efficient caproic acid synthesis in the fourth-batch APM since a higher level of caproic acid was detected. Additionally, we found succinate semialdehyde and acetyl-CoA as two important links in multiple metabolic pathways that drain into the butanoate metabolism. The detected enrichment of acetyl-CoA acetyltransferase (*Swol_1934*) in the butanoate metabolism from *Syntrophomonas* suggests that rare bacterial groups may play important roles in SFB production. One challenge in SFB production is how to reduce lactic acid accumulation in pit mud since an excess of lactic acid is related to the increase in ethyl lactate that deteriorates SFB quality (21). In our study, the detected decrease in the abundance of d-lactate dehydrogenase (*ldhD*) in pyruvate metabolism is desirable in SFB production as this enzyme can generate lactic acid from pyruvate (44). The detected higher pyruvate and lower lactic acid levels in the fourth-batch APM samples agreed with the prediction. Taken together, the model provides a first systems-level overview of the effects of microbial succession on SFB quality.

### Conclusion.

This multi-omics study allowed the identification of taxon-specific functions and metabolic pathways that significantly differ during artificial pit mud maturation. The results revealed key linkages between several members of the prokaryotic community and metabolites and proteins in the artificial pit mud. These candidate microbial species, proteins, and metabolites may provide targets for predicting the quality of APM and its fermentation state, which will be helpful for further optimization of APM culture technique and improve the quality of strong-flavor baijiu. The results also emphasize the importance of longitudinal, multi-omics study designs to unravel the effects of brewing microbial communities and their contribution to strong-flavor baijiu production.

## MATERIALS AND METHODS

### Materials.

APM samples were obtained from new fermentation cellars from an SFB-producing enterprise in Luzhou, Sichuan, China (105°27′13.80″ E, 28°53′6.90″ N). APM samples from eight cellars were selected for metabolomic analysis, and physicochemical properties, flavor compounds, 16S rRNA gene amplicons, and metaproteomes were analyzed from three of these. APMs were sampled at the initial state of SFB brewing and at the end of the first four brewing batches from September 2017 to December 2018. The brewing period of each batch was 90 days. APM samples (50 g) were collected from all corners and the center of the cellar walls, combined, and stored at –80°C. For each brewing batch, approximately 100 ml of SFB was collected into glass bottles, sealed, and stored at 4°C.

The APM for the cellars was prepared by mixing fresh soil with natural mature pit mud that had been in use for 50 years, wheat bran, and soybean meal (13:1:1:0.5, wt/wt/wt/wt) to form a solid matrix. The matrix was incubated with a starter culture (10:1, wt/wt) and appropriate amounts of SFB, tap water, and yellow water (a by-product formed during SFB brewing). Finally, the mixture was covered with polyester film and incubated at ambient temperature (20 to 34°C, with an average of 24°C) for 34 days (from 28 July 2017 to 30 August 2017). The starter culture preparation is described in detail in our previous study (45).

### APM physicochemical properties.

The APM moisture was measured using a dry/wet weight measurement method after drying the APM at 105°C for 3 h. The pH was determined in a 1:2.5 APM/water (wt/vol) slurry using a pH meter (PB10; Sartorius, Gottingen, Germany). The concentration of humus was determined as described by Mehlich (46). The concentrations of organic matter, total N, total P, total K, NH_4_^+^, available P, and available K were measured as described previously (47).

### SFB flavor compounds.

Flavor compounds in SFB samples were extracted as described previously (48) and quantified using a Clarus 500 gas chromatograph (PerkinElmer, Waltham, MA) equipped with flame ionization detectors (FIDs) and a 50-m by 0.25-mm by 0.2-μm CP-Wax 57 CB column (Agilent, Santa Clara, CA) as described previously (28). The compounds were quantitated by comparison with the internal standards 2-methyl-2-butanol, pentyl acetate, and 2-ethylbutyric acid (Sigma-Aldrich, Shanghai, China).

### 16S rRNA gene amplicon sequencing and qPCR.

DNA was extracted from the APM using a FastDNA Spin Kit for Soil (MP Biomedicals, Solon, OH) according to the manufacturer’s instructions. For amplicon sequencing, the V3-V4 regions of the 16S rRNA genes were amplified using the primers 338F and 806R (49). PCR amplification, library preparation, and Illumina MiSeq sequencing were done as described previously (28).

The raw sequences were processed using the QIIME platform (version 1.9.1). Briefly, the forward and reverse reads were joined and assigned to samples based on the barcode sequences and then truncated by removing the barcode and primer sequences. Quality filtering of the joined sequences was performed, and sequences with lengths of <200 bp, ambiguous bases, and mean quality scores of ≤20 were discarded. The quality-filtered sequences were binned into OTUs at a 97% similarity level using the clustering program VSEARCH (version 1.9.6) against the Silva 132 database (https://www.arb-silva.de/) (50). The OTUs were assigned to taxa using the Ribosomal Database Project (RDP) Classifier at an 80% confidence level (51). The Chao1 richness index and Shannon diversity index were calculated using Mothur (version 1.30.1 [http://www.mothur.org/]) (52). Comparative community analysis at the OTU level was done using PCA with STAMP (version 2.1.3 [http://kiwi.cs.dal.ca/Software/STAMP]) (53). Metabolic functions of the microbial communities were predicted using PICRUSt (version 1.0.0 [http://picrust.github.com/picrust/]) with KO classification (54).

*Clostridia* and *Lactobacillus* were quantified with qPCR using a 7900HT real-time PCR system (Applied Biosystems, Foster City, CA) and primers SJ-F/SJ-R (55) and Lac1/Lac2 (56) for *Clostridia* and *Lactobacillus*, respectively. Amplification was done in 20-μl reaction mixtures containing SYBR Green Realtime PCR Master Mix (Transgen, Beijing, China), 2 pmol of each primer, template DNA, and distilled water, as described previously (22).

### Protein preparation.

For protein extraction, 1 g of APM sample was ground in liquid nitrogen with 10% (wt/wt) polyvinylpolypyrrolidone (PVPP). The proteins in the samples were then extracted using the citrate/SDS-phenol (C/S-P) method (57). Briefly, the proteins were recovered by citrate (0.25 M, pH 8) and SDS buffer (1.25% [wt/vol] SDS, 0.1 M Tris-HCl, pH 6.8, 20 mM dithiothreitol [DTT]), extracted with phenol (pH 8), and precipitated by methanol and cold acetone. Subsequently, 100 μg of protein was trypsin digested as described previously (58). For the PRM experiments, the digested peptides were desalted on 100-mg C_18_ reverse-phase SPE columns according to the manufacturer’s instructions (Waters, Milford, MA).

### TMT-MS proteomics.

TMT labeling was carried out using a TMT six-plex kit (Thermo Fisher Scientific, San Jose, CA) according to the manufacturer’s instructions. The first-batch APM samples were labeled with 126, 127, and 128 mass tags, the fourth-batch APM samples were labeled with 129, 130, and 131 tags, and the samples were mixed at equal amounts. Labeled peptides were fractionated using an Agilent 1100 HPLC (high performance liquid chromatograph) and lyophilized. The lyophilized peptide fractions were dissolved into 1 μg μl^−1^ using 0.1% formic acid (vol/vol), and 1 μl of the peptide solution was analyzed using liquid chromatography-tandem MS (LC-MS/MS) with an EASY-nLC 1200 system and a Q-Exactive hybrid quadrupole-orbitrap mass spectrometer (Thermo Fisher Scientific). The detailed methods for peptide fractionation and LC-MS/MS analysis are provided in Text S1 in the supplemental material.

10.1128/mSystems.00829-19.7 TEXT S1 Detailed methods. Download Text S1, DOCX file, 0.02 MB. Copyright © 2020 Liu et al. 2020 Liu et al. This content is distributed under the terms of the Creative Commons Attribution 4.0 International license.

Peptides and proteins were identified with the Sequest HT search engine using Proteome Discoverer (version 2.2; Thermo Scientific) software, and the raw MS/MS data were compared against a database created by collating all UniProt entries corresponding to organisms (class level) with an abundance of >1% in the 16S rRNA gene amplicon sequencing data from the first- and fourth-batch APM samples. We used the setting “enable protein group” in Proteome Discoverer to group redundant proteins that share sets of identified peptides. The proteins within a group are scored according to the number of unique peptides, the number of peptide spectrum matches (PSMs), and the sequence coverage. The highest-scoring protein of a group was selected as the representative protein (master protein). Database searches were performed using the following parameters: sample type, TMT six-plex (peptide labeled); Cys alkylation, iodoacetamide; digestion, trypsin; instrument, Q-Exactive. The global false-discovery rate (FDR) was ≤0.01, and protein groups considered for quantification required at least one unique peptide. Proteins with fold change values of ≥2 or ≤0.5 (*P < *0.05) were considered differential. GO enrichment and KEGG pathway analysis were performed using OmicsBean software (http://www.omicsbean.cn), using Escherichia coli K-12 MG1655 as a reference organism.

### Target analysis by PRM-MS proteomics.

The protein expression levels obtained using TMT-MS analysis were confirmed by quantifying the expression levels of 15 selected proteins by PRM-MS analysis with an EASY-nLC 1200 system and a Q-Exactive mass spectrometer (Thermo Fisher Scientific). The detailed LC-MS/MS analysis methods are described in Text S1. MS raw data from data dependent analysis (DDA) were compared against the database used for TMT-MS proteomics using Proteome Discoverer (version 2.2; Thermo Scientific). The FDR was set to 0.01 for proteins and peptides. Quantitative data processing was done with Skyline software (version 4.1.0) (59) using the following parameters: precursor charges, 2; ion charges, 1 and 2; ion type, y and b; product ion selection, 3 ions; special ions, N-terminal to proline; ion match tolerance, 0.05 *m/z*; precursor mass analyzer, Orbitrap; isotope peaks included for MS filtering, count; MS resolving power at 200 *m/z*, 60,000; acquisition method selected for MS/MS filtering, targeted; MS/MS resolving power at 200 *m/z*, 30,000.

### Metabolomic analysis of APM.

Untargeted metabolomic analysis to identify the differential metabolites between the first- and fourth-batch APM samples was done using GC-MS and LC-MS. GC-MS analysis was performed using a 7890B gas chromatograph (Agilent) coupled to a 5977A mass selective detector (Agilent). LC-MS analysis was performed on an Acquity UPLC system (Waters, Milford, MA) coupled with a Xevo G2-XS quadrupole time-of flight (QToF) mass spectrometer (Waters). The detailed sample preparation and MS analysis methods are described in Text S1.

GC-MS raw data were converted to computable document format (CDF) using ChemStation (version E.02.02.1431; Agilent). Data processing was done using Chroma TOF (version 4.34; Leco, St. Joseph, MI). Metabolites were annotated by searching the databases of Kind et al. (60). LC-MS raw data were processed using Progenesis QI (Waters). Metabolites were annotated using the human metabolome database (HMDB) (61) and LIPID MAPS (62). Statistical tests were done using SIMCA (version 14.0; Umetrics, Umeå, Sweden). Differential metabolites were identified using an OPLS-DA model; variables with a VIP value of >1 and a Student's *t* test *P* value of <0.05 were considered differential. The quality of the OPLS-DA models was verified by the typical cross-validation procedure of leaving one-seventh of the samples out of each round and then assessing the model. The pathways of the metabolites were identified using MBROLE, version 2.0 (63), based on the KEGG database using Escherichia coli K-12 MG1655 as a reference organism.

### Statistical analysis.

Statistical analysis was performed with SPSS, version 20.0, software for Windows (SPSS, Inc., Chicago, IL). Pearson’s correlation coefficient analysis was applied to investigate the associations between APM prokaryotic community, physicochemical properties, and SFB flavor compounds. Differences in the APM physicochemical properties, SFB flavor compounds, prokaryotic diversity indices, and the relative abundance of the taxa and KOs among groups were tested by one-way analysis of variance (ANOVA) with Duncan’s multiple-range tests.

### Data availability.

The sequencing data were submitted to the Sequence Read Archive of the NCBI database under BioProject PRJNA577744 (BioSample accession numbers SAMN13037336 to SAMN13037350). The mass spectrometry proteomics data were submitted to the iProX database (64) (accession number IPX0001834000, https://www.iprox.org//page/SCV017.html?query=IPX0001834000) and ProteomeXchange (accession number PXD016140, http://proteomecentral.proteomexchange.org/cgi/GetDataset?ID=PXD016140).
