# Supplementary figures and images for: Structural and Functional Changes in Prokaryotic Communities in Artificial Pit Mud during Chinese Baijiu Production
Source: mSystems. 2020 Mar 24;5(2):e00829-19. doi: 10.1128/mSystems.00829-19 (PMC7093824; doi:10.1128/mSystems.00829-19)

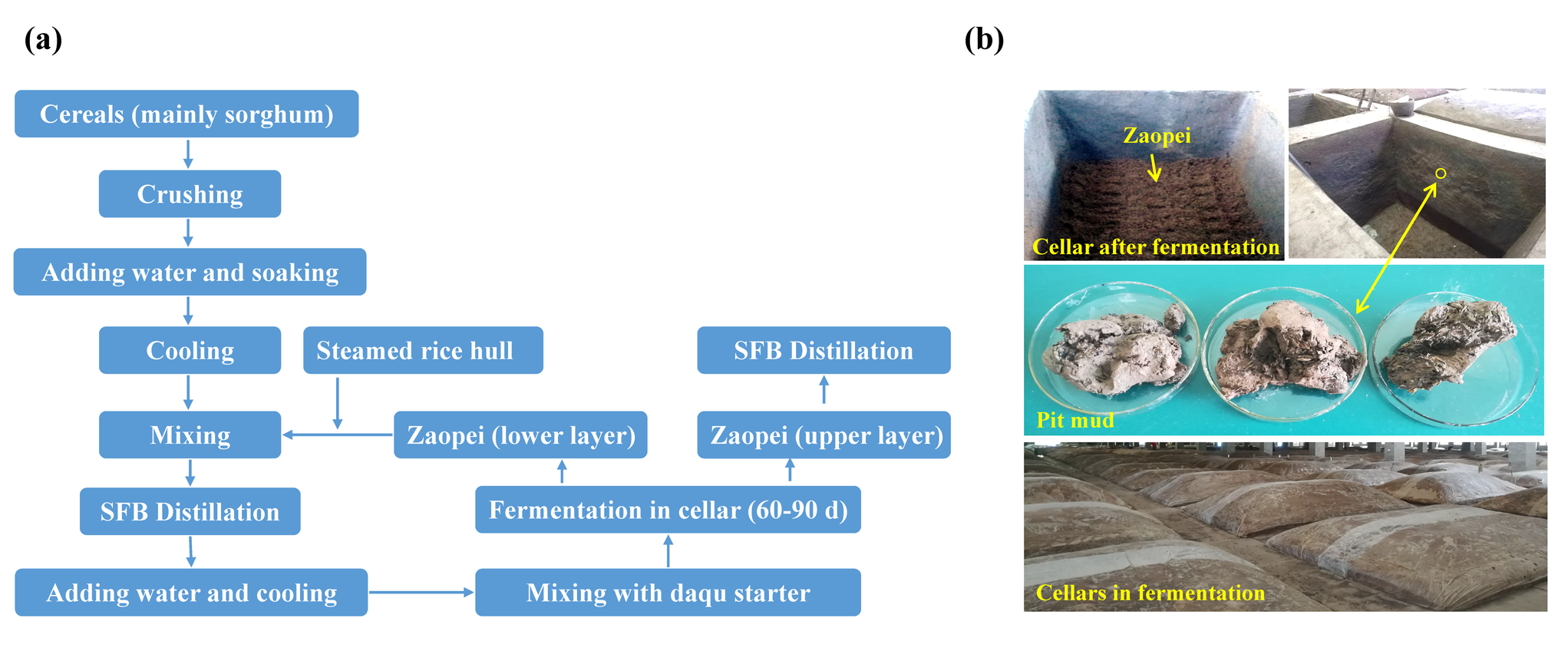

Supplement: FIG S1 [file mSystems.00829-19-sf001.tif]
