# Supplementary material for: Structural and Functional Changes in Prokaryotic Communities in Artificial Pit Mud during Chinese Baijiu Production
Source: mSystems. 2020 Mar 24;5(2):e00829-19. doi: 10.1128/mSystems.00829-19 (PMC7093824; doi:10.1128/mSystems.00829-19)
Supplement: TEXT S1 [file mSystems.00829-19-s0001.docx]

**TMT-MS Proteomics**

APM proteins were analyzed by TMT-MS proteomics. The detailed methods for peptide fractionation and LC-MS/MS analysis are presented below.

*Reversed phase liquid chromatography fractionation.* Reversed phase liquid chromatography separation was performed on an 1100 HPLC system (Agilent, Santa Clara, CA, USA) using a Zorbax Extend C18 column (5 μm, 150 mm × 2.1 mm, Agilent). Mobile phases A (2% acetonitrile, v/v, pH 10.0) and B (90% acetonitrile, pH 10.0) were used for a gradient. The solvent gradient wasset as follows: 0-8min, 98% A; 8.00-8.01 min, 98-95% A; 8.01-38 min, 95-75% A; 38-50 min, 75-60% A; 50-50.01 min, 60-10% A; 50.01-60 min, 10% A; 60-60.01 min, 10-98% A; 60.01-65 min, 98% A. Tryptic peptides were separated at a fluent flow rate of 300 μL/min and ten combined fractions were vacuum-dried until LC-MS/MS analysis.

*LC-MS/MS analysis*. All analysis were performed using a Q-Exactive (Thermo Fisher Scientific, San Jose, CA, USA) equipped with a Nanospray Flex source (Thermo Fisher Scientific). The lyophilized peptide fractions were loaded and separated by an Acclaim PepMap RSLC C18 (15 cm × 75 µm) on an EASY-nLC 1200 system (Thermo Fisher Scientific). Mobile phases A (0.1% formic acid) and B (80% acetonitrile containing 0.1% formic acid) were used for a linear gradient. The flow rate was 300 nL/min and linear gradient was 90 min (0-55 min, 8% B; 55-79 min, 30% B; 79-80 min, 50% B; 80-90 min, 100% B). Full MS scans were acquired in the mass range of 300-1,600 m/z with a mass resolution of 70,000 and the AGC target value was set at 1e6. The top ten intense peaks in MS were fragmented with higher-energy collisional dissociation (HCD) of 32. MS/MS spectra were obtained with a resolution of 17,500, with an AGC target of 2e5 and a maximum injection time of 80 ms. Dynamic exclusion was set for 15 sand run under positive mode.

**PRM-MS Proteomics**

The protein expression levels determined by TMT analysis were confirmed by quantifying the expression levels of 15 selected proteins using PRM-MS analysis. The detailed methods of LC-MS/MS analysis are shown below.

*LC-MS/MS analysis*. A Q-Exactive (Thermo Fisher Scientific) coupled with EASY-nLC 1200 system (Thermo Fisher Scientific) was used for both spectral library construction and PRM experiments. The peptide were loaded on an Acclaim PepMap RSLC C18 (15 cm × 75 µm) with a flow rate of 400 nL/min (buffer A: 2% acetonitrile containing 0.1% formic acid, buffer B: 95% acetonitrile containing 0.1% formic acid). The gradient was performed as follows: 0-10 min, 6% B；10-55 min, 24% B；55-79 min, 40% B；79-90 min, 80% B; 90-100 min, 1% B. The peptides were transferred to the gaseous phase with positive ion electrosprayionization at 2.1 kV.

For the data dependent analysis (DDA) mode, the MS was taken at a resolution of 70,000 with a scan mass range of 350 to 1,550 m/z, a target (AGC) of 1e6, and the maximum injection fill time was 50 ms. The ten most intense ions were isolated for MS/MS fragmentation. MS/MS (m/z 100-1,500) was employed at a resolution of 17,500, a target AGC of 2e5, and the maximum injection fill time was 50ms. Fragmentation was performed with higher-energy collisional dissociation (HCD) mode of 30% and a dynamic exclusion duration of 15 s. For PRM, precursors were targeted in a 1.2 m/z isolation window around the m/z of interest. Precursors were fragmented in HCD mode with normalized collision energy (NCE) of 25. The MS was performed at 70,000 resolution, an AGCtarget of 1e6, and a maximum injection time of 50 ms. MS/MS was performed at 17,500 resolution, an AGC target of 2e5, and themaximum injection time was 50 ms.

**Metabolomic analysis**

*Sample preparation*. The APM sample (1 g) was transferred to a 2 mL EP tube. Two small steel balls were added to the tube. Next, 20 μL internal standard (2-chloro-l-phenylalanine in methanol, 0.3 mg/mL) and 1 mL extraction solvent with 50% methanol were added to each sample. Samples were stored at -80 °C for 2 min and then subjected to grinding at 60 HZ for 2 min. All mixtures of each sample were transferred to 15 mL EP tubes, and the residual materials were transferred with 1 mL 50% methanol, and this step was repeated twice. The mixture was centrifuged at 4 °C at 8000 r/min for 10 min, and 2.5 mL of the supernatant was transferred to 5 mL EP tubes and then freeze-dried. Subsequently, the freeze-dried samples were dissolved in 500 μL 50% methanol, vortexed for 60 s, and then transferred to 1.5 mL EP tubes. The mixture was centrifuged at 12000 rpm, 4 °C for 10 min. A QC sample was prepared by mixing aliquots of all samples (a pooled sample).

For GC-MS analysis, 300 μL supernatant was transferred to a glass sampling vial and vacuum-dried at room temperature. Next, 80 μL of methoxylamine hydrochloride (dissolved in pyridine, 15 mg/mL) was added. The resultant mixture was vortexed vigorously for 2 min and incubated at 37 °C for 90 min. Next, 80 μL of BSTFA (with 1% TMCS) and 20 μL n-Hexane were added into the mixture, vortexed vigorously for 2 min, and then derivatized at 70 °C for 60 min. The samples were placed at ambient temperature for 30 min before analysis. For LC-MS analysis, the supernatants (200 μL) from each tube were collected using crystal syringes, filtered through 0.22 μm microfilters, and transferred to LC vials for analysis.

*GC-MS analysis.* GC-MS analysis was performed using a 7890B gas chromatograph (Agilent) coupled to a 5977A mass selective detector (Agilent). A 1 μL aliquot of derivatized sample was injected into a DB-5MS fused-silica capillary column (30 m × 0.25 mm × 0.25 μm, Agilent) using split mode at a ratio of 15:1. Helium (> 99.999%) was used as the carrier gas at a constant flow rate of 1 mL/min through the column. The oven temperature was initially held at 60 °C, then increased to 125 °C at a rate of 8 °C /min, to 210 °C at a rate of 5 °C /min, to 270 °C at a rate of 10 °C/min, to 305 °C at a rate of 20 °C/min, and finally maintained at 305 °C for 5 min. The temperatures of the MS quadrupole and ion source (electron impact) were set to 150 °C and 230 °C, respectively. MS detection was achieved using 70 eV electron and full-scan mode (m/z 50-500). QC samples were injected at regular intervals (every 4 samples) throughout the analytical run.

*LC-MS analysis.* LC-MS analysis was performed on an Acquity UPLC system (Waters, Milford, MA, USA) coupled with Xevo G2-XS QTof mass spectrometer (Waters). A 2 μL aliquot of supernatant was chromatographed on an Acquity UPLC BEH C18 column (2.1 × 100 mm, 1.7 μm, Waters) using a constant flow rate of 400 μL/min. The gradient elution system consisted of buffer A (HPLC water containing 0.1% formic acid) and buffer B (acetonitrile containing 0.1% formic acid) and separation was achieved using the following gradient: 0 min, 1% B; 1 min, 5% B; 2 min, 30% B; 3.5 min, 60% B; 7.5 min, 90% B; 9.5 min, 100% B; 12.5 min, 100% B; 12.7 min, 1% B and 16 min, 1% B. Argon (99.999%) was used as the collision-induced dissociation gas. The other MS parameters were as follows: scan type, MS^E^ centroid, a low-energy scan (CE 6eV), and a high-energy scan (CE ramp 20-35eV) to fragment the ions; scan rate, 0.1 s；capillary voltages, +3 kV (positive mode) and -2 kV (negative mode), reference capillary voltage, 2.5 kV; cone voltage, 40 V; source offset, 80 V; source temperature, 120 °C; desolvation gas temperature, 450 °C; desolvation gas flow, 800 L/h, and cone gas flow, 50 L/h. QC samples were injected at regular intervals (every 4 samples) throughout the analytical run.
